# How to improve the environmental impact in haemodialysis: small actions, big changes

**DOI:** 10.1093/ckj/sfae407

**Published:** 2024-12-20

**Authors:** María Dolores Arenas Jiménez, Julia Audije-Gil, Rodrigo Martínez, Natalia Martín Vaquero, Miquel Gómez, Jesús Portillo, Gerard Pereda, David Hernán Gascueña, Brett Duane, Marta Sanjuan, José Luis Fernández Martín, Fabiola Dapena, Alberto Ortiz, Marta Arias

**Affiliations:** Unidad de Investigación, Fundación Renal, Madrid, Spain; Unidad de Investigación, Fundación Renal, Madrid, Spain; Departamento de Nefrología e Hipertensión, IS-Fundación Jiménez Díaz, Madrid, Spain; Unidad de Investigación, Fundación Renal, Madrid, Spain; Servicio de Nefrología, Hospital Clínic, Barcelona, Spain; Unidad de Investigación, Fundación Renal, Madrid, Spain; Servicio de Nefrología, Hospital Clínic, Barcelona, Spain; Unidad de Investigación, Fundación Renal, Madrid, Spain; Public Dental Hea, Trinity College Dublin, Dublin, Ireland; Unidad de Investigación, Fundación Renal, Madrid, Spain; Bone and Mineral Research Unit, Instituto de Investigación Sanitaria del Principado de Asturias, Oviedo, Spain; Unidad de Investigación, Fundación Renal, Madrid, Spain; Departamento de Nefrología e Hipertensión, IS-Fundación Jiménez Díaz, Madrid, Spain; Servicio de Nefrología, Hospital Clínic, Barcelona, Spain

**Keywords:** dialysis, green nephrology, key performance indicators, kidney replacement therapy, sustainability

## Abstract

**Background:**

The burden of chronic kidney disease is increasing, driven by population aging and the increase in risk factors. In-centre haemodialysis (HD), which accounts for most of the environmental impact of kidney replacement therapy, is a power-hungry, water-hungry and a waste-generating intervention.

**Methods:**

Here we characterize the environmental impact of 20 HD centres over 5 years and its modulation by operational changes (centre size, opening days, geographic location, type of water treatment plant and disinfection, dialysis modalities such as HD versus haemodiafiltration, seasonal variations and the impact of various corrective actions throughout time).

**Results:**

Average water and energy consumption per session were lower in larger centres compared with smaller ones, as well as in centres that operated daily compared with those that opened three times a week (both *P* < .05). Seasonality, which depends on the geographic region, had a marked influence (*P* < .001) on water and energy consumption, as does the choice of water treatment plant (*P* < .001). Actions taken in certain centres significantly reduced energy consumption up to 30%, plastic waste up to 65% and hazardous waste up to 63%.

**Conclusion:**

Annual monitoring must be performed to detect variations and plans must be designed to reduce the environmental impact. As it is not possible to reduce HD water and energy consumption to zero, implementing targeted actions offers a promising strategy for reducing the environmental burden.

## Graphical Abstract

**Graphical Abstract:**
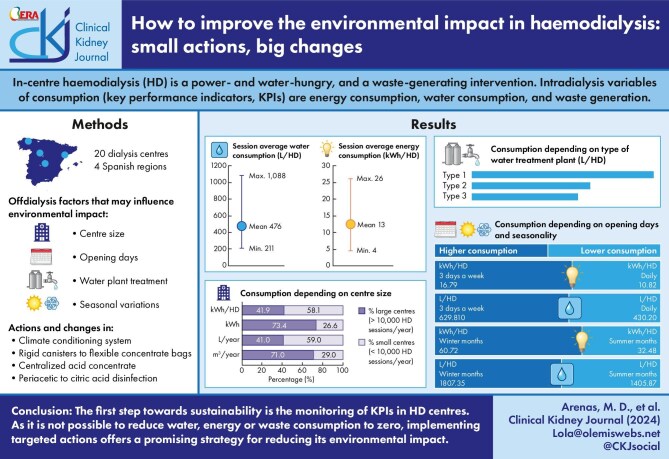

KEY LEARNING POINTS **What was known:**

- Kidney replacement therapy (KRT) centres consume a large amount of resources such as water and electricity and generate a large amount of waste.
- In-centre haemodialysis (HD) accounts for ≈70% of all KRT. An HD session is equivalent to the daily water consumption of 3.5–4 people and the average daily electricity consumption of a centre is equivalent to that of 2.5–3 households (9 kWh/day/household).
- In order to design and implement tailored actions based on their specific characteristics it is necessary to assess the environmental impact of each individual HD centre.

**This study adds:**

- This study provides data from a 5-year follow-up in 20 HD centres in different geographic regions, considering the influence of different operational characteristics (centre size, opening days, geographic location, type of water treatment plant and disinfection, dialysis modalities such as HD versus hemodiafiltration, seasonal variations and the impact of various corrective actions throughout time).
- Average water and energy consumption per session were lower in larger HD centres, as well as in centres that operated daily. Seasonality has a marked influence on water and energy consumption, as does the choice of water treatment plant. The latter is a key factor in water and energy consumption.
- Certain actions diminished environmental impact: improving climate conditioning systems decreased energy consumption by 6–30% changing from acid concentrate canisters to flexible bags or changing from acid concentrate canisters/bags to centralized tanks reduced plastic waste by 62–65% and switching from peracetic to citric acid disinfectant reduced chemical waste by 63%.

**Potential impact:**

- This study encourages HD centres to measure their environmental impact and establish their own standards in key performance indicators, such as electricity consumption (kWh/session), water consumption (l/session) and waste generation (kg/session).
- Manageable actions, such as individualization and selection of climate-appropriate systems, can optimize energy efficiency and reduce the overall environmental impact of HD. Along with annual monitoring to design action plans, these small actions can do their bit to make the world a more sustainable place.

## INTRODUCTION

Healthcare activities are responsible for roughly 5–6% of total carbon dioxide (CO_2_) emissions in developed countries [1]. Within nephrology, nearly 4 million people worldwide need kidney replacement therapy (KRT), with in-centre haemodialysis (HD) accounting for ≈70% of all KRT [2]. The environmental burden of in-centre HD is elevated due to high water and energy consumption [3], as well as hazardous and non-hazardous waste generation [4]. There is a need to raise public awareness and inform policymakers about the importance of ensuring the environmental sustainability of KRT [5, 6] by prioritizing the optimization of building infrastructure and management systems [7, 8].

In this regard, there are established sets of measurable scores, known as key performance indicators (KPIs), that evaluate the environmental burden of in-centre HD [9]. KPIs are independent, easy to measure and individually evaluated for each HD facility based on their characteristics (size, location, HD techniques available).

Most of the information recently published on in-centre HD sustainability consists of systematic reviews with theoretical tips or guidelines [10–13]. However, there is an unmet need for implementation of these tips and guidelines using a holistic approach that expands beyond HD treatment itself into broader elements such as optimized logistics, supply chains and facility adequacy with improved efficiency [14–16].

The aim of this study was to understand the environmental impact of in-centre HD using the three most commonly used KPIs [9]: energy consumption, water consumption and waste generation, over a 5-year multicentre follow-up. Additionally, the study evaluated how individual characteristics (centre size, working days, geographical location, type of water treatment plant, suppliers) influence their environmental impact and assessed the impact of corrective actions.

## MATERIALS AND METHODS

### Study design

This work was a multicentre retrospective study conducted over 5 years (2019–2023) in 20 HD centres across four Spanish geographic regions: Madrid (9 centres, M1–M9), Catalonia (1 centre, B1), Galicia (6 centres, G1–G6) and Castile and Leon (4 centres, C1–C4). Seventeen centres were operational throughout the 5-year study period, with two centres starting in 2020 (one in Galicia and one in Madrid) and one in 2023 (in Madrid). Not all centres measured all indicators throughout the 5 years. All centres are certified according to the International Organization for Standardization 14001 standard.

### Factors and actions associated with environmental impact

Modifiable factors that may influence environmental impact were analysed: centre size (<10 000 sessions/year for small centres and >10 000 sessions/year for larger ones); operating schedule (thrice-weekly or daily, consisting of 6 days/week, except on Sundays); type of commercial provider plant (anonymized as type 1, 2 and 3); water treatment plant disinfection methods (only chemical carried out 4 times/year or combined thermal and chemical disinfection, performed twice a week for 30–40 min, except for centre B1, which used 1 session/week for 90 min); dialysis modalities, such as conventional HD versus haemodiafiltration online (HDF-OL), and HD session duration; and non-modifiable factors such as seasonality. Regarding water consumption, the flow used was 500 ml/min.

The impact of the following corrective actions was evaluated: changes in the climate conditioning system (in centres C1–C4); change from rigid dialysate acid concentrate canisters to flexible bags (in C1, C2, B1, M2 and M5–M8); change from dialysate acid concentrate canisters/flexible bags to centralized acid concentrate tanks (in G4, M1 and M6–M8) versus centres that used centralized acid since their opening; and change of disinfectant from peracetic acid to citric acid (G4 and M3).

### Variables

**Figure 1:**
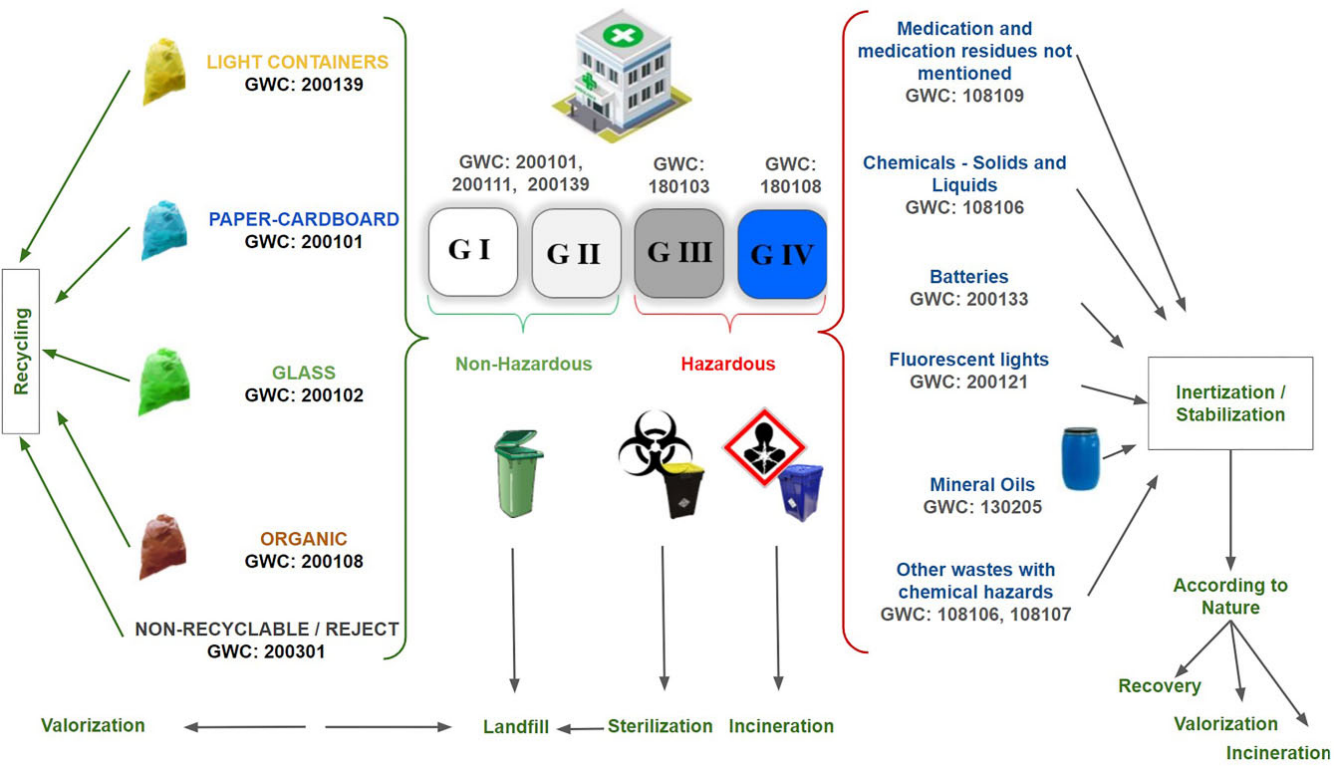
Classification of waste generated in haemodialysis (HD). Adapted from Arias-Guillén et al. (2024) [13].

The variables studied included monthly and annual consumption and indicators: number of HD sessions, water consumption [cubic metres (m^3^); litres per HD session], energy consumption [kilowatt-hours (kWh), megawatt-hours (MWh), kWh/HD session] and hazardous and non-hazardous waste [kilograms (kg), tonnes (t), kg/HD session]. The most common hazardous wastes in HD are biomedical waste and chemical waste, and the most common non-hazardous wastes were plastic waste (total plastics and plastics from acid concentrate, citric acid or bleach), paper waste and non-recyclable general waste (Fig. 1).

The measurements for the variables water consumption (in m^3^) and energy consumption (in kWh) were obtained from invoices, where the absolute consumption data for both parameters are recorded. In both cases, the total water consumption of the centre was included (for dialysis treatment, cleaning and disinfection of equipment, general hygiene for the centre, restrooms, etc.), as well as the total electricity consumption (for dialysis equipment, water treatment plant, lighting, climate control, computers, disinfection, etc.). In the case of waste, the measurement depends on the type of waste. Hazardous waste, according to Spanish legislation, is measured at the final treatment plant (in kg), which sends monthly or annual reports with full traceability linked to the organization that generated the waste. Non-hazardous waste is estimated based on the weight of bags for each type of waste produced, except in the case of plastic, where estimations are made using the weight of various empty containers and the consumption figures for each store.

### Statistical methods

Results were presented as absolute annual values and normalized by HD session. The average water and energy consumption and waste generation per HD patient per year, together with the Spanish and European HD population burden, were estimated considering a usual thrice-weekly schedule (3 sessions/week over 52 weeks/year equals 156 sessions annually).

Univariate analyses were performed to compare the influence of each factor and/or implementing action (average comparison before and after implementation). The collected data were analysed using SPSS Statistics for Windows version 29.0.2.0 (IBM, Armonk, NY, USA). The significance level was set at *P* < .05.

## RESULTS

Data presented here correspond to a total of 919 059 HD sessions performed over 5 years in 20 geographically diverse HD centres. While the patient population evolved over time, this number of HD sessions would be equivalent to providing chronic HD to 1179.6 persons continuously for 5 years. The overall combined water consumption over 5 years was 311 027.2 m^3^, energy consumption was 7155.8 MWh and waste production was 628.6 t [49.2 t of hazardous waste (45.8 t of biomedical and 3.34 t of chemical waste) and 579.4 t of non-hazardous waste (88.9 t of plastics, 3.1 t of paper and 487.4 t of non-recyclable waste). The main contributor to plastic waste was dialysate acid concentrate container plastic (78.6 t over 5 years).

The annual average number of HD sessions performed in all centres was 9882.4 ± 6278.3 (range 756–22 436). Given the heterogeneity of centre size and operating procedures, Table 1 presents water and energy consumption and waste generation as the total annual average and as relative average per HD session.

**Table 1:** Global consumption and waste generation data of all 20 centres throughout the study period (2019–2023).

Characteristics	Annual average	HD session average
Number of HD sessions	9882.4 ± 6278.3 (756.0–22 436.0)	–
Water consumption	3747.3 ± 1915.1 (479.7–7449.0) m^3^	476.5 ± 194.8 l (210.8–1087.6)
Energy consumption (kWh)	115 416.0 ± 74147.0 (18 339.0–295 650.0)	12.6 ± 5.9 (4.4–26.3)
Hazardous waste		
Biomedical (kg)	790.2 ± 953.0 (18.6–4172.5)	0.079 ± 0.047 (0.025–0.210)
Chemical (kg)	42.8 ± 45.8 (0.4–183.7	0.006 ± 0.004 (2.27 × 10^−4^–0.250)
Non-hazardous waste		
Total plastic (kg)	946.1 ± 765.8 (40.6–4137.0)	0.128 ± 0.079 (0.012–0.256)
Acid concentrate plastic (kg)	883.6 ± 763.0 (39.0–4038.2)	0.123 ± 0.080 (0.008–0.247)
Citric acid plastic (kg)	29.6 ± 22.4 (0.3–83.1)	0.004 ± 0.003 (0.001–0.016)
Bleach plastic (kg)	18.9 ± 14.7 (0.8–81.1)	0.003 ± 0.001 (2.17 × 10^−4^–0.009)
Paper (kg)	70.0 ± 62.6 (13.0–350.0)	–
Non-recyclable (kg)	27 076.2 ± 22 550.1 (1514.3–70 358.0)	2.1 ± 0.9 (0.1–3.4)

Data are expressed as mean ± standard deviation (range).

### Estimating the national and European burden

Water and energy consumption and waste generation derived from providing HD for a single person over 1 year are estimated in Table 2. The average water consumption per patient per year was 74 326.2 l, energy consumption was 1962.5 kWh and waste generation was 376.4 kg. In 2021, there were 26 683 prevalent HD cases in Spain [17] and 166 707 in Europe [18]. This means the average water consumption of the Spanish population per year was 1 983 246.0 m^3^, energy consumption was 52 364.9 MWh and waste generation was 10 044.2 t. The average water consumption of the European HD population per year was 12 390 697.8 m^3^, energy consumption was 327 159.2 MWh and waste generation was 62 753.2 t.

**Table 2:** Estimation of Spanish and European HD population consumption and waste generation in 1 year.

Characteristics	Single HD patient/year	Spanish HD population/year	European HD population/year
Number of HD patients	1	26 683	166 707
Number of HD sessions	156	4 162 548	26 006 292
Water consumption	74 326.2 l	1 983 246.0 m^3^	12 390 697.8 m^3^
Energy consumption	1962.5 kWh	52 364.9 MWh	327 159.2 MWh
Hazardous waste			
Biomedical	12.3 kg	328.8 t	2054.5 t
Chemical	0.9 kg	25.0 t	156.0 t
Non-hazardous waste			
Total plastic	20.0 kg	532.8 t	3328.8 t
Acid concentrate plastic	19.2 kg	512.0 t	3198.8 t
Citric acid plastic	0.6 kg	16.7 t	104.0 t
Bleach plastic	0.5 kg	12.5 t	78.0 t
Paper	–	–	–
Non-recyclable	322.9 kg	8616.5 t	53 833.0 t

### Factors associated with environmental impact

Centre size, operating schedule, type of water treatment plant, dialysis modalities (such as conventional HD versus HDF-OL) and HD session duration and season were associated with the environmental impact of in-centre HD. As regards the size of the centre, 6 centres (40%) were considered large (>10 000 HD sessions/year) and 12 (60%) were small. Water and energy consumption were higher in large versus small centres when expressed as an annual average: 5756.1 ± 1067.2 versus 2353.5 ± 800.6 m^3^ and 164 405.1 ± 69 452.9 versus 59 669.8 ± 20 429.6 kWh (*P* < .001), respectively. However, the average annual water and energy consumption per HD session were lower in large versus small centres: 377.9 ± 123.7 versus 542.8 ± 206.6 l (*P* < .001) and 10.7 ± 6.4 versus 14.8 ± 4.2 kWh (*P* = .002), respectively (Table 3).

**Table 3:** Factors that influence water and energy consumption per HD session.

	Water consumption (l/HD)	*P*-value	Energy consumption (kWh)	*P*-value
Media/HD session	476.5 ± 194.8	–	12.6 ± 5.9	–
Centre size				
Large	377.9 ± 123.7	**<.001**	10.7 ± 6.4	**.002**
Small	542.8 ± 206.6		14.8 ± 4.2	
Days open per week				
6	430.2 ± 180.8	**<.001**	10.8 ± 5.7	**<.001**
3	629.8 ± 160.8		16.8 ± 3.9	
Type of water treatment plant				
1	682.9 ± 124.1	**<.001**	13.8 ± 6.3	**<.001**
2	444.1 ± 82.4		18.9 ± 6.5	
3	398.9 ± 182.8		9.8 ± 2.9	
Disinfection				
Only chemical	516.0 ± 190.3	**.042**	12.9 ± 5.4	0.726
Chemical + thermal	428.4 ± 191.8		12.3 ± 6.4	
Season				
Winter (December–February)	453.9 ± 181.6	.123	15.4 ± 8.4	**<.001**
Summer (July–September)	466.3 ± 181.3		10.8 ± 6.4	

Significant values in bold.

Sixteen centres (80%) dialysed 6 days/week and 4 (20%) just 3 days/week. The average annual water and energy consumption per centre was higher in those that operated 6 days/week than in those that operated only 3 days/week: 4282.7 ± 1848.3 versus 1944.0 ± 547.5 m^3^ (*P* < .001) and 141 331.5 ± 73 081.4 versus 52 067.0 ± 15 183.3 kWh (*P* < .001), respectively. However, the average annual water and energy consumption per HD session was lower in centres that operated 6 versus 3 days/week: 430.2 ± 180.8 versus 629.8 ± 160.8 l (*P* < .001) and 10.8 ± 5.7 versus 16.8 ± 3.9 kWh (*P* < .001), respectively (Table 3).

Regarding the water treatment plant, 10 centres (50%) had type 1, 6 (30%) had type 2 and 4 (20%) had type 3. Water consumption differed for different water treatment plants. Average values per HD session were 682.9 ± 124.1 l for type 1, 444.1 ± 82.4 l for type 2 and 398.9 ± 182.8 l for type 3 (*P* < .001) (Table 3). Significant differences in average energy consumption per HD session were also found between water treatment plants: 13.8 ± 6.3 kWh for type 1, 18.9 ± 6.5 kWh for type 2 and 9.8 ± 2.9 kWh for type 3 (*P* < .001).

As to disinfection, 9 centres (45%) had thermal–chemical disinfection. These centres used less average water per HD session (428.4 ± 191.8 versus 516.0 ± 190.3 l; *P* = .042). However, energy consumption was similar (average per HD session: 12.3 ± 6.4 versus 12.9 ± 5.4 kWh; *P* = .726) (Table 3).

In relation to the dialysis modality, centres with more than half of the sessions in conventional HD modality consumed less water per HD session compared with those with more than half in HDF-OL, but these differences were not statistically significant (432.7 ± 190.2 l/HD in those with <50% HDF-OL versus 510.8 ± 219.8 l/HD in those with >50% HDF-OL; *P* = .118). Nevertheless, significant differences were found in electricity consumption: 11.2 ± 4.0 l/HD in those with <50% HDF-OL versus 13.1 ± 5.8 l/HD in those with >50% HDF-OL (*P* = .006).

The average session duration for all patients was ≈4 h (223 ± 10 min). There were no patients with 8-h sessions or nocturnal dialysis. A moderately positive correlation was observed between the duration of the HD session (minutes) and water consumption (*r* = 0.49; *P* = .021), as well as energy consumption (*r* = 0.50; *P* = .008).

**Figure 2:**
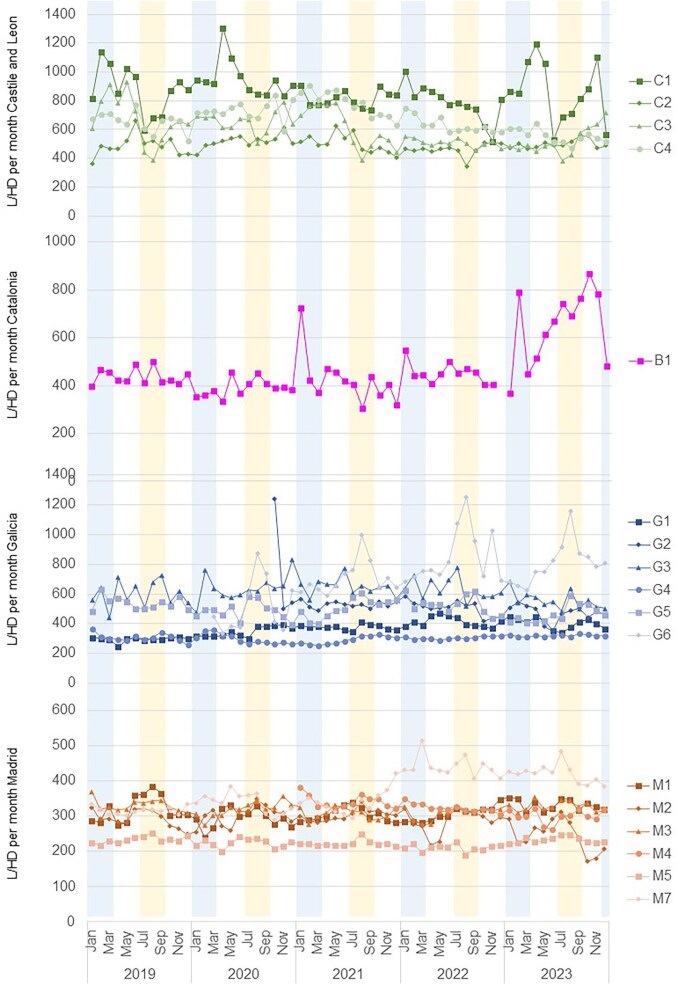
Water consumption (L) per haemodialysis (HD) session from January 2019 to December 2023 in the centres of Castile and Leon, Catalonia, Galicia, and Madrid. Winter months are shaded in blue and summer months in yellow.

**Figure 3:**
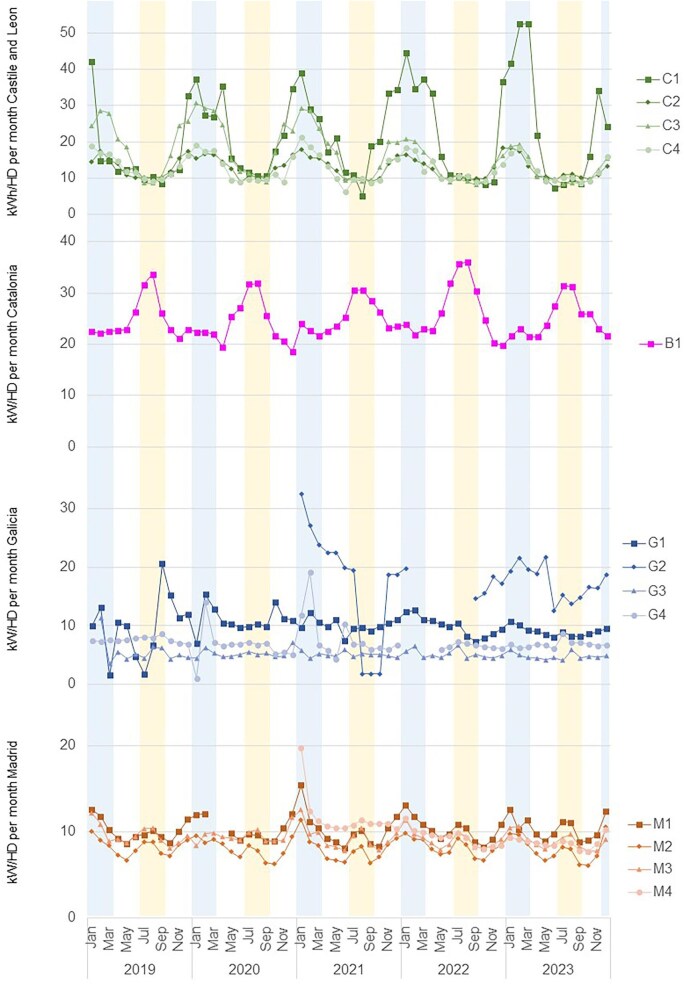
Energy consumption (kWh) per haemodialysis (HD) session from January 2019 to December 2023 in the centres of Castile and Leon, Catalonia, Galicia, and Madrid. Winter months are shaded in blue and summer months in yellow.

Seasonal variations in energy and water consumption were observed between and within the regions, as well as between centres (Table 3). The average 3-month water use per session was higher in summer (466.3 ± 181.3 l/HD session) than in winter (453.9 ± 181.6 l/HD session), although not significantly (*P* = .123). In contrast, energy consumption per HD session was higher in winter than in summer months (15.4 ± 8.4 kWh/HD session in winter versus 10.8 ± 6.4 kWh/HD session in summer; *P* < .001) (Figs. 2 and 3). However, these variations depended on the geographic region: in Catalonia energy consumption was higher in summer, in Castile and Leon it was higher in winter and in Madrid there was no seasonal variability.

### Actions and environmental consequences

#### Changes in the climate conditioning system

The climate control system was adjusted in centre C1 to improve patient comfort and in C2 and C3 to reduce costs and enhance insulation. Centre C1 significantly increased its average energy consumption per HD session, from 16.0 ± 0.0 to 22.3 ± 0.9 kWh (*P* = .005) (Fig. 3). In contrast, centres C2 and C3 significantly reduced their energy consumption from 13 ± 0.2 to 12.2 ± 0.2 kWh (*P* = .023) and from 18.6 ± 1.1 to 12.7 ± 0.7 kWh (*P* = .003), resulting in reductions of 5.8% and 31.6% in energy consumption, respectively.

#### Change from dialysate acid concentrate canisters to flexible bags

**Figure 4:**
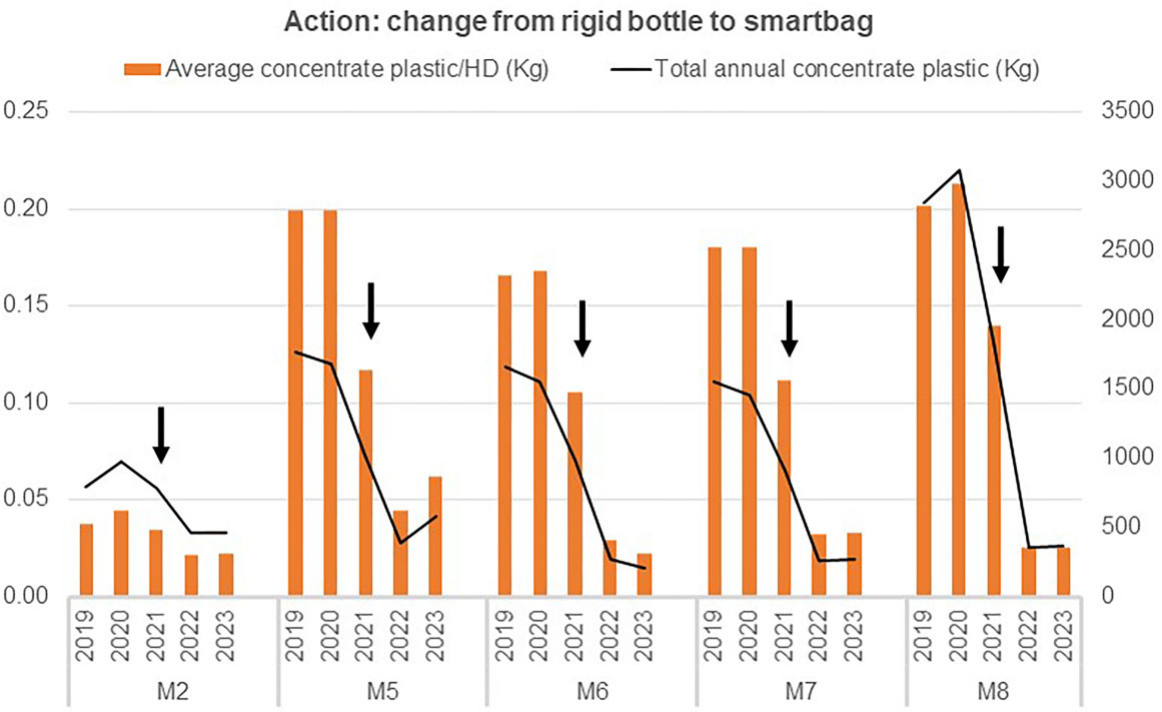
Impact on plastic consumption of changing from rigid canister to flexible bags in five centres from Madrid (M2, M5 to M8). Action implemented in July 2021 (black arrows).

Eight centres (40%; C1, C2, M2 and M5–M8) changed from rigid acid canisters to flexible bags, which reduced the annual plastic waste assimilable to urban waste from acid concentrate by 61.6%. The average annual plastic waste from acid concentrate decreased from 155.6 ± 86.8 kg (range 43.8–336.5) to 59.7 ± 36.7 kg (21.8–167.1) and the average per HD session decreased from 0.2 ± 0.03 kg (0.09–0.21) to 0.12 ± 0.06 kg (0.03–0.20) (both *P* < .001) (Fig. 4).

#### Change from acid concentrate canisters/flexible bags to centralized acid concentrate (tanks)

In 2019, centralized dialysis fluid was present in 2 of 16 centres (12.5%; B1 and M3); by the end of the study period in 2023, this number had increased to 8 of 20 centres (40%; G4, B1, M1, M3–M6 and M8).

**Figure 5:**
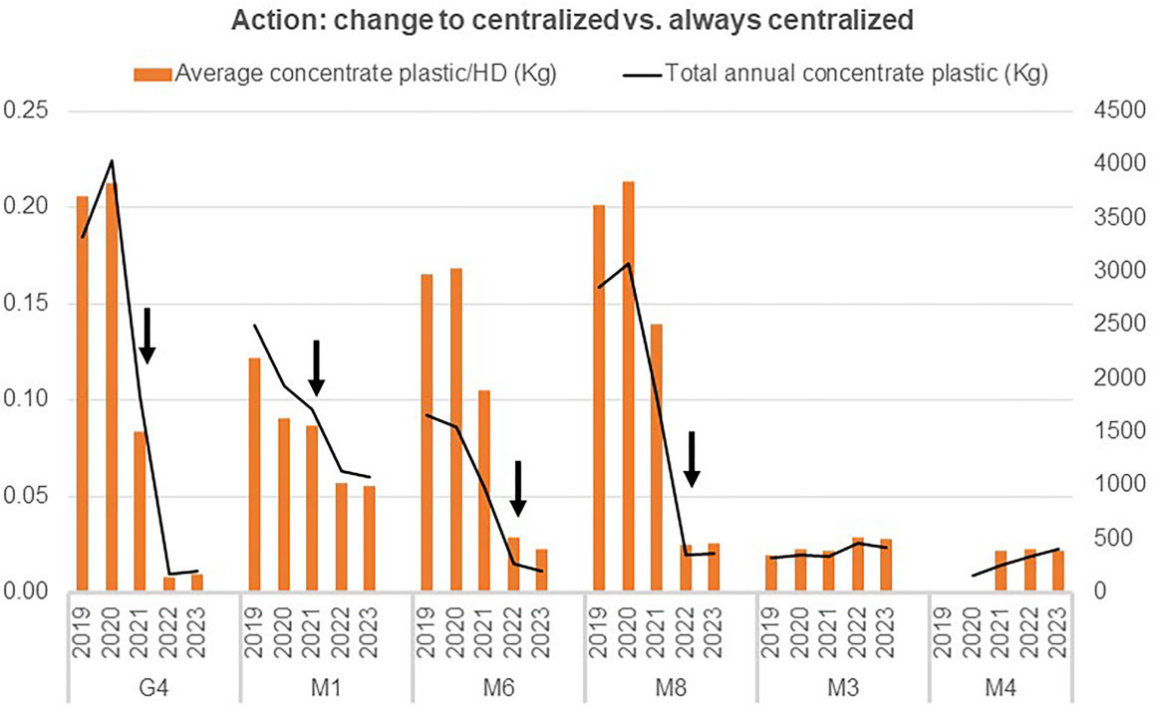
Impact on plastic consumption of changing to centralized (centre G4 from Galicia in April 2021; M5 in August 20, M6 in February 2022, and M8 from Madrid in March 2022; black arrows) versus remaining on centralized dialysate acid concentrate (centre M3 and M4 from Madrid).

The change from rigid containers/flexible bags to centralized acid concentrate tanks reduced the average annual plastic waste by 65.107% (*P* < .001), from 146.9 ± 110.3 kg (range 29.7–503.6) to 51.3 ± 38.5 kg (14.6–152.7) (Fig. 5).

In centres using rigid containers, the average annual plastic waste per HD session from acid concentrate was 0.16 ± 0.06 kg (range 0.01–0.24). In centres using flexible bags, it was 0.11 ± 0.07 kg (0.02–0.20), and in centres with centralized systems, it was 0.03 ± 0.02 kg (0.008–0.08) (*P* < .001) (Fig. 5).

#### Change in disinfection fluid

**Figure 6:**
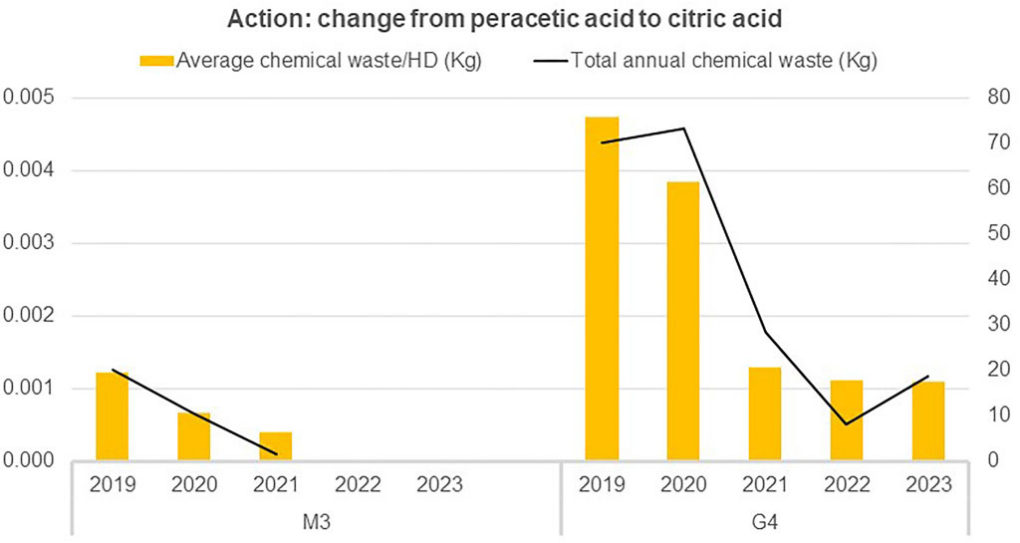
Impact on hazardous waste of changing disinfectant from peracetic acid to citric acid in two centres of Galicia (G4) and Madrid (M3). Action implemented in July 2021, black arrows.

In 2021, two centres (10%; G4 and M3) transitioned the type of disinfectant fluid used for the water treatment system and monitors from peracetic acid to citric acid. This change resulted in a reduction of total annual chemical waste by 62.6% (from 2.62 × 10^−3^ ± 1.98 × 10^−3^ to 9.81 × 10^−4^ ± 3.95 × 10^−4^ kg; *P* = .098) (Fig. 6).

## DISCUSSION

To the best of our knowledge, this is the first multicentre study to provide not only a long-term follow-up of KPIs of HD centres, but also to emphasize the environmental implications of the size of the centre, operating schedule, geographic location and type of water treatment plant. It also highlights different sustainability actions that may achieve a reduction of 30–65% in consumption and waste generation.

Prior studies have estimated the carbon footprint (measured as CO_2_ equivalents) of HD [19, 20]. It is anticipated that a European Union funded project (KIT-NEWCARE [21]) will use the life cycle assessments methodology, which includes carbon footprinting. However, the precise calculation of greenhouse emissions are often sophisticated, while the follow-up of water and energy consumption and waste generation is a manageable step towards estimating environmental burdens. Nonetheless, most HD centres around the globe neither monitor indicators nor have sustainability strategies in place (including educational interventions) to reduce their impact [6–8, 22].

In 2020, Bendine *et al.* [23] reported a 13-year period in France in which a systematic reduction of water use per HD session (52.3%; 801 to 382 l/HD session), energy consumption (29.6%; from 23.1 to 16.3 kWh/HD session) and waste generation (37.3%; 1.8 to 1.1 kg/HD session) was achieved through water treatment system improvements and dialysis unit equipment and building updates. Another study in the USA in 15 large centres [24] documented 1-year average water and energy consumption per HD session of 600 ± 200 l and 25.6 ± 7.6 kWh, respectively, as well as 1.2 ± 0.2 kg of biomedical waste and 13.4 ± 4.1 kg of landfill waste.

HD is often cited as a power- and water-hungry therapy [25]. On average, an HD session requires ≈500 l of water [26] and 12.0–19.6 kWh of electrical energy [27, 28]. Our data generally aligns with these quantities, and our results further highlight some factors that may influence water and energy consumption per HD session, such as centre size, operating schedule, seasonality and type of water treatment plant. The use of indicators (l, kWh, kg) scaled to the number of HD sessions performed allows for comparing centres with different characteristics and activities. Both average water and energy consumption indicators were lower in larger centres compared with smaller ones, as well as in centres that operated daily compared with those with a thrice-weekly schedule. Even with fewer dialysis sessions per year, smaller centres and those operating thrice weekly have a proportionally higher water and energy consumption per session because the water treatment plant expends energy and uses water during start-up, priming, rinsing cycles and disinfection [29], regardless of the number of sessions performed.

In this study, centres with a higher proportion of HDF-OL had greater water and energy consumption, although the difference in water consumption was not significant. High-dose HDF-OL has shown benefits over conventional high-flux HD in terms of reduced risk of death from any cause [30], although it could be associated with greater consumption. Recent studies [31] involving >26 000 patients have demonstrated that optimising the HDF-OL prescription by adjusting dialysis flow to blood and ultrafiltration flow maximises the filtration fraction, which can reduce water and dialysate consumption while increasing solute clearance and maintaining similar dialysis doses. Regarding dialysis fluid consumption, the flow rate used in the majority of centres was 500 ml/min, so it was not possible to study the impact of different flow rates on water consumption, although presumably the higher the flow rate, the higher the consumption.

Seasonality, which depends on the geographic region where the centre is located, has a marked influence on consumption. In the case of water, a possible reason for this seasonality could be the influence of rainy or dry periods on the quality, hardness and conductivity of the water [13]. In the case of energy consumption, our results show that individualization, climate system improvement and investment in adequate insulation can optimize energy efficiency and reduce the overall environmental impact (energy consumption reduction of up to 5.84% and 31.6%, respectively, for centres C2 and C3). In this regard, Madrid centres showed less variability between seasons despite the regional climate, precisely due to having better insulation since their construction. In centre C1, the changes were made to improve patient comfort, without considering the impact on energy consumption. A balance should be achieved between a comfortable temperature for patients and professionals and climate efficiency and energy consumption [26].

Choosing efficient technology in the water plant is crucial to optimizing the centre water consumption. Current systems reject between 50–70% of the source water [29, 32], being the design of the water treatment technology a highly influential factor in the use of water [14]. Implementing advanced and innovative technologies can reduce residual water waste generation by up to 20%. However, newer systems often have a reduced membrane lifespan [3, 27].

Looking to the future, it is essential to reduce or reuse water rejected by reverse osmosis [11]. At least 108 m3 per year could be saved by reusing the treatment water [32]. Potential applications for this residual water waste include facilities cleaning, agriculture, aquaponics, and horticulture, among others [33, 34].

Regarding water treatment plant electricity consumption, thermal disinfection is considered one of the most energy-intensive processes in a dialysis session [32]. However, our results did not disclose significant differences between chemical versus chemical plus thermal disinfection. The balance must be achieved between water saving (with significantly lower consumption with thermal disinfection) and the reduction of hazardous waste. One possible measure proposed in the literature is to evaluate the frequency of disinfections, in order to save water and electricity [35].

In reference to waste generation, HD generates on average between 1.5-8 kg of waste per session [4, 36]. Specifically, it is estimated that 38% of that waste is plastic, and usually of single use [29]. It is therefore vitally important to implement measures to reduce waste generation.

The present study identified actions can increase HD sustainability by reducing plastic and hazardous waste. The use of acid concentrate flexible bags and the use of centralized acid concentrate can reduce plastic waste generation by significant percentages of 61.6% and 65.1%, respectively. On the one hand, the flexibility of bags reduces packaging volume and weight compared to canisters [36]. On the other, the use of centralized fluid reduces acid waste itself, cardboard waste or the need for boxes, plastic packaging and energy for transporting materials [7, 19].

As for disinfection, this procedure is unavoidable, but the focus must be towards more sustainable and biodegradable alternatives that offer the same clinical results. According to our results switching from peracetic acid disinfection fluid to citric acid reduced annual chemical waste by 62.6%, although further research is required. It should be noted, however, that citric acid containers are generally more easily recyclable than peracetic acid containers, as they are classified as hazardous waste.

Finally, staff training is essential for optimal waste management and classification since proper selection reduces the environmental impact [14, 32]. The reduction and proper segregation of waste should be a primary goal in HD centres, as the amount of waste will naturally increase with the growing number of kidney patients. The main proposal is to make use of the 5 R rule: reduce, reuse, recycle, rethink and research [10].

With all the above, the first step towards sustainability is the monitoring of KPIs in HD centres. The second step being the urgent need to implement actions and innovative approaches. While it is not possible to reduce consumption to zero, implementing targeted actions offers a promising strategy for reducing its environmental footprint.

However, certain limitations should be acknowledged. First, the inclusion of only 20 geographically limited centres, all located in Spain, may restrict the generalizability of the study. Additionally, the financial cost implications were not addressed. As a future perspective, one parameter not included in the study was the use of dialysate-to-blood flow adjustment monitors, which were implemented in only three centres and not for all patients (only for those with low fistula flow rates). Thus, the preliminary results were inconclusive, showing no significant impact on water consumption. Therefore, future research will explore the effects of autoflow monitors, as adjustments to dialysate flow may influence water usage. Besides the use of autoflow, it would also be interesting to explore the impact of incremental HD initiation (only 2 sessions per week as long as there is residual renal function), as presumably, a lower number of sessions would favour a lower consumption of resources, as well as a lower generation of waste.

In conclusion, factors influencing energy efficiency and water consumption include the size of the centre (lower consumption per HD session in larger centres), operating schedules (lower consumption in centres that operate 6 days per week), choice of water treatment plant and seasonality (energy and water consumption being lower in summer months). Individualization and selection of climate-appropriate systems and most sustainable commercial providers can optimize energy efficiency and reduce the overall environmental impact of HD.

The environmental impact of HD may be reduced by improving climate conditioning systems (energy consumption reduction of 6–30%), changing from acid concentrate canisters to flexible bags (≈62% annual reduction in plastic waste), changing from acid concentrate canisters/bags to centralized tanks (≈65% annual reduction in plastic waste) and switching from peracetic acid disinfection liquid to citric acid (≈63% reduction in annual chemical waste).

## Data Availability

The data underlying this article will be shared upon reasonable request to the corresponding author.
